# Toll-like receptor 4 stimulation with monophosphoryl lipid A ameliorates motor deficits and nigral neurodegeneration triggered by extraneuronal α-synucleinopathy

**DOI:** 10.1186/s13024-017-0195-7

**Published:** 2017-07-04

**Authors:** Serena Venezia, Violetta Refolo, Alexia Polissidis, Leonidas Stefanis, Gregor K. Wenning, Nadia Stefanova

**Affiliations:** 1 Division of Neurobiology, Department of Neurology, Innsbruck Medical University, Innsbruck, Austria; 2 Laboratory of Neurodegenerative Diseases, Division of Basic Neurosciences, Biomedical Research Foundation of the Academy of Athens, Athens, Greece

**Keywords:** α-synuclein, Toll-like receptor, Inclusion pathology, Neuroinflammation, Monophosphoryl lipid A

## Abstract

**Background:**

Alpha-synuclein (α-syn) aggregation represents the pathological hallmark of α-synucleinopathies like Parkinson’s disease (PD), dementia with Lewy bodies (DLB), and multiple system atrophy (MSA). Toll-like receptors (TLRs) are a family of highly conserved molecules that recognize pathogen-associated molecular patterns and define the innate immunity response. It was previously shown that TLR4 plays a role in the clearance of α-syn, suggesting that TLR4 up-regulation in microglia may be a natural mechanism to improve the clearance of α-syn. However, administration of TLR4 ligands could also lead to dangerous adverse effects associated with the induction of toxic inflammatory responses. Monophosphoryl lipid A (MPLA) is a TLR4 selective agonist and a potent inducer of phagocytosis which does not trigger strong toxic inflammatory responses as compared to lipopolysaccharide (LPS). We hypothesize that MPLA treatment will lead to increased clearance of α-syn inclusions in the brain of transgenic mice overexpressing α-syn in oligodendrocytes under the proteolipid protein promoter (PLP-α-syn mouse model of MSA), without triggering toxic cytokine release, thus leading to a general amelioration of the pathology.

**Methods:**

Six month old PLP-α-syn mice were randomly allocated to four groups and received weekly intraperitoneal injections of MPLA (50 or 100 μg), LPS or vehicle. After a 12-week treatment period, motor behavior was assessed with the pole test. Brains and plasma samples were collected for neuropathological and immunological analysis.

**Results:**

Chronic systemic MPLA treatment of PLP-α-syn mice led to increased uptake of α-syn by microglial cells, a significant motor improvement, rescue of nigral dopaminergic and striatal neurons and region-specific reduction of the density of oligodendroglial α-syn cytoplasmic inclusions in the absence of a marked systemic inflammatory response.

**Conclusion:**

Our findings demonstrate beneficial effects of chronic MPLA treatment in transgenic PLP-α-syn mice. MPLA appears to be an attractive therapeutic candidate for disease modification trials in MSA and related α-synucleinopathies.

**Electronic supplementary material:**

The online version of this article (doi:10.1186/s13024-017-0195-7) contains supplementary material, which is available to authorized users.

## Background

α-synucleinopathies are neurodegenerative disorders characterized by the presence of abnormal α-synuclein (α-syn) inclusions in the brain, predominantly in neurons in Parkinson’s disease (PD) and dementia with Lewy bodies (DLB), and in oligodendrocytes in multiple system atrophy (MSA) [1–5]. α-syn is a 140-amino-acid protein widely expressed in the brain especially in the presynaptic nerve terminals. In physiological conditions, α-syn is naturally present unfolded in the cytoplasm [6] and its monomeric state is stabilized by intramolecular interactions that prevent its oligomerization [7, 8]. Misfolding and intracellular aggregation of α-syn are crucial in the pathogenesis of PD, DLB and MSA. Recent studies show that cell-to-cell transmission of toxic α-syn is possible and represents an important step leading to spreading of α-syn inclusions, subsequent neurodegeneration and progression of the disease [9–11]. The mechanisms responsible for this spreading may involve release of the protein in the extracellular space via vesicle-mediated exocytosis, packaging into exosomes or leakage through damaged membranes [12, 13]. Therefore, to intervene in the clearance of a-syn would mean also to interfere with the progression of the disease.

Neuroinflammation occurs in parallel with the progression of the disease in α-synucleinopathies [14]. Microglial activation may trigger the release of deleterious cytokines and chemokines, but it may also clear toxic α-syn via increased phagocytosis and proteolytic degradation, which may lead to neuroprotection. Toll-like receptors (TLRs) are a family of highly conserved molecules that define the innate immune response [15] and are present on the surface of microglial cells. Recent observations show up-regulation of TLRs in α-synucleinopathies like MSA and PD [16–19]. We found that TLR4 mediates α-syn-induced microglial phagocytic activity, pro-inflammatory cytokine release and ROS production [20]. Furthermore, we demonstrated both in vitro and in vivo that TLR4 mediates the uptake and clearance of α-syn by microglia. Transgenic mice overexpressing α-syn in oligodendrocytes under the proteolipid protein promoter (PLP-α-syn mice) lacking functional TLR4 showed markedly elevated brain levels of α-syn linked to increased loss of nigral neurons and augmented motor disability as compared to PLP-α-syn mice with functional TLR4 [21]. These findings raise the possibility that stimulation of microglial cells with TLR4 agonists may accelerate α-syn clearance and result in beneficial effects in α-synucleinopathies. Therefore, we investigated herein the efficacy of chronic administration of the nonpyrogenic TLR4 agonist monophosphoryl lipid A (MPLA) in PLP-α-syn mice - a transgenic mouse model of MSA. MPLA is a chemically detoxified form of lipopolysacharide (LPS) which shows unique immunomodulatory properties [22]. MPLA, in comparison to LPS, is broadly reported to induce reduced release of cytotoxic mediators which makes it a commonly applied vaccine adjuvant [23–27].

Our current results show that MPLA treatment, in the absence of a marked systemic inflammatory response, increases the uptake of α-syn by microglia, and ameliorates the characteristic phenotype of PLP-α-syn transgenic mice by reducing intracellular α-syn aggregation, rescue of SNc dopaminergic neurons and improvement of motor deficits.

## Methods

### Animals and treatment

PLP-α-syn mice expressing human wild-type α-syn under the control of an oligodendroglia-specific PLP promoter to model the typical pathology of MSA [28], were maintained on a light/dark cycle of 12 h (lights on at 6 am) with free access to food and water at the Animal Facility of the Medical University of Innsbruck. Six month old male and female PLP-α-syn mice, were randomly allocated to four groups and received over a period of 12 weeks a weekly, intraperitoneal injection of either MPLA (50 μg and 100 μg, vac-mpls, MPLAs Vaccigrade, Invivogen, San Diego, CA, USA), LPS (3 μg, Sigma-Aldrich, Vienna, Austria) or vehicle. Importantly, the MPLA preparation used here (vac-mpls) is synthetic lipid A from *E. coli*, serotype R515, i.e. a pure monophosphoryl lipid A compound produced by chemical synthesis. This preparation activates TLR4 but does not activate TLR2 reflecting its high purity (http://www.invivogen.com/mplas-vaccigrade). All the experiments were done according to the EU and the Austrian legislation and with permission of the Ethics Board at the Federal Ministry of Science and Research, Austria (permission BMWFW-66.011/0122-WF/V/3b/2014). All analyses were done by a researcher who was blinded to the treatment of the animals.

### Motor behavior

After 12 weeks of treatment, the pole test was employed to evaluate the fine motor coordination, balance, and bradykinesia. Each mouse was placed at the top of a vertical pole (50 cm) with head up and the time needed to turn and orient the body downwards (Tturn) and the time to reach the bottom with all four paws (Ttotal) was measured over three trials. The best performance was kept for statistical analysis.

### Histological analysis

Two weeks after the last injection, mice were deeply anesthetized with an overdose of thiopental and then perfused intracardially with phosphate buffered saline (PBS, pH 7.4, Sigma-Aldrich, Vienna, Austria) followed by ice-cold 4% paraformaldehyde (PFA, pH 7.4, Sigma-Aldrich, Vienna, Austria). Brains were rapidly removed, post-fixed in 4% PFA overnight at 4 °C and then cryoprotected in 30% sucrose solution in PBS at 4 °C. Afterwards, brains were frozen using 2-methylbutan (Merck, Darmstadt, Germany) and stored at −80 °C until further processing. Whole brains were cut in 40 μm thick sections on a freezing microtome (Leica, Nussloch, Germany).

Immunohistochemistry was performed according to a standard protocol for free-floating sections and the following antibodies were used: monoclonal mouse anti-tyrosine hydroxylase (TH; Sigma-Aldrich, Vienna, Austria), mouse anti-dopamine and cyclic AMP-regulated phosphoprotein (DARPP32, BD Transduction Laboratories), rabbit anti-phosphorylated α-syn (pS129; Abcam, UK), rat anti-human α-syn (15G7, Enzo, Lörrach, Germany), anti-aggregated human α-syn (5G4, Linaris, Dossenheim, Germany), and anti-Iba1 (Abcam, UK). Sections were then incubated with the appropriate biotinylated secondary antibody followed by Vectastain ABC reagent (Vector Laboratories, Burlingame, CA, USA) and 3,3′-diaminobenzidine (Sigma-Aldrich, Vienna, Austria) to visualize the immunohistochemical binding sites. Stained sections were mounted on slides, dehydrated and coverslipped with Entellan (Merck, Darmstadt, Germany). For immunofluorescence, suitable IgGs conjugated with Alexa 488 or Alexa 594 (Life Technologies, Eugene, OR, USA) were applied. Sections were coverslipped with mounting medium (Dianova, Hamburg Germany).

### Image analysis

Stereological analysis was performed using the Nikon E-800 microscope equipped with Nikon digital camera DXM 1200 and Stereoinvestigator Software (MicroBrightField Europe e.K., Magdeburg, Germany) as described previously [29]. The number of TH- and DARPP-32- positive neurons in the SNc and striatum respectively was assessed by the optical fractionator. The density of pS129, 15G7, and 5G4-positive inclusions was assessed in SNc, striatum and cerebellum with meander scan using the same software.

Three-dimensional stacks were acquired with an SP8 confocal microscope (Leica Microsystems, Wetzlar, Germany) using a HC PL APO CS2 63×, 1.3 NA glycerol immersion objective. Imaging was performed using white light laser with excitation lines for Alexa 488 at 498 nm and for Alexa 594 at 590 nm. Fluorescence emission was detected in sequence 1 from 503 to 576 nm (Alexa 488) and in sequence 2 from 594 to 742 nm (Alexa 594). Images were acquired using the Leica LAS X 3.1.1 acquisition software (Leica Microsystems). Image deconvolution was performed using Huygens Professional software (Scientific Volume Imaging, Hilversum, Netherlands). The Ortho Slicer function was used to define spatial co-localization of signals in 3D. For the analysis of the percentage of phagocytic cells under different conditions, random Iba-1-positive cells were acquired in z-stacks by a researcher blinded to the treatment, and further analysed for the presence or absence of α-syn in the cytoplasm.

### Phagocytosis assay

BV2 immortalized murine microglial cells were seeded at a density of 15 × 10^3^ per well in a 24-well plate with 500 μl of DMEM supplemented with 10% fetal calf serum (FCS) and 2 nM L-Glutamine. Two days later the medium was exchanged and 3 nM soluble α-syn monomers, or 3 nM preformed fibrils of α-syn prepared according to previously published protocol [30], or the same volume of medium were added. After 1 h incubation at 37 °C, cells were treated with vehicle, MPLA or LPS and further incubated for another 4 h. Phagocytic activity was determined using fluorescent microspheres (1 μm, Invitrogen, San Diego, CA, USA) and compared to the untreated controls. After incubation for 30 min with the fluorescent microspheres, cells were fixed with 4% PFA, and phagocytic cells were counted in eight random fields. All measurements were repeated in four separate biological replicates. The results were expressed as percentage of phagocytic cells per treatment.

### Cytokine/chemokine levels

Blood samples were collected by facial vein puncture, centrifuged to separate the plasma and frozen at −80 °C. Hemibrain tissue was snap-frozen after PBS perfusion and stored at −80 °C until analysis. Tissue homogenates from hemibrains were prepared in RIPA-lysis buffer containing 65 mM Tris-base, 150 mM NaCl, 1% Triton-X, 0.25% sodium deoxycholate, 1 mM EDTA, and a mix of phosphatase and protease inhibitors (“phosSTOP” and “Complete, mini, EDTA-free,” Roche Applied Science). BCA Protein Assay Kit (Pierce #23225, ThermoScientific, Waltham, MA USA) was used for determining the total protein concentration. To measure the concentration of cytokines we used the ProcartaPlex® Multiplex Immunoassay (eBioscience, Waltham, MA USA) that uses the Luminex technology (multi-analyte profiling beads) to enable the simultaneous detection and quantitation of multiple cytokines and chemokines per sample. All samples were measured in duplicate and the mean values of the two readouts were calculated and used in subsequent statistical analysis. Data for the analysis are presented as pg cytokine/chemokine per mg total protein for the brain lysates or pg/ml plasma sample.

### Alpha-synuclein quantification with ELISA and Western blot

The distribution of different species of α-syn in the brains of PLP-α-syn mice was analyzed by Western blot. Hemibrain tissue was collected after PBS perfusion and stored at −80 °C until analysis. Tissue was homogenized in Triton-X-100 (TX) extraction buffer (150 mM NaCl, 50 mM Tris pH 7.6, 1% Triton-X-100, 2 mM EDTA) containing protease and phosphatase inhibitors. The lysate was sonicated and then centrifuged (120,000 × g for 60 min at 4 °C) and the supernatant was collected (TX soluble fraction). The pellet was then washed three times with 1 M PBS/1% TX, centrifuged (13,000 × g for 15 min) and re-suspended in SDS extraction buffer (150 mM NaCl, 50 mM Tris pH 7.6, 1% Triton-X-100, 0.5% Na-deoxycholate, 1% SDS), sonicated, left on ice for 30 min and centrifuged (120,000×g for 60 min at 4 C) and the supernatant was collected (SDS soluble fraction). The pellet was washed three times with 1 M PBS/1% SDS and centrifuged (13,000 × g for 15 min) and then dissolved in 8 M urea/SDS (5%) solution, mixed and boiled for 5 min three times, centrifuged (120,000×g for 60 min at 18 °C) and the supernatant was collected (Urea fraction). The samples were run on 12% SDS-PAGE gels. Primary antibodies included antibodies against α-syn [rabbit monoclonal phosphor-α-syn (pS-129), 1:500; Abcam, EP1536Y], [human-specific monoclonal 4B12, 1:1000; Genetex, GTX21904], [monoclonal syn-1, 1:1000; BD Biosciences, 619,787), [polyclonal C20, 1:1000; Santa Cruz, sc-7011-R] and β-actin [1:2000; Cell Signalling Technology, 8H10D10] as a loading control. The intensity of the immunoreactive bands was estimated by densitometric quantification using ImageJ (relative density, RD).

The total level of α-syn was measured with ultra-sensitive ELISA. The sandwich ELISA for total α-syn as previously described [31] was adapted for measurement in brain lysates. 50 μl of brain lysate (TX-fraction: 1:5000 dilution; SDS-fraction: 1:500 dilution; urea fraction was undetectable with this method due to background interference of the buffer) or recombinant α-syn (as standard), diluted in TBST/BSA (10 mM Tris-Cl, pH 7.6, 100 mM NaCl, 0.1% Tween-20 and 1% BSA) were measured. Data are expressed as μg of α-syn versus mg of total protein estimated with the Bradford method (Biorad protein assay).

### Statistical analyses

All statistical analyses were conducted using the software Graph-Pad Prism 5 (Graphpad Software, San Diego, CA). The mean ± S.E.M was used to present the results. One-way analysis of variance (ANOVA) with post-hoc Bonferroni test was used to compare the groups if not indicated otherwise. A *P*-value <0.05 was considered statistically significant. Correlations were studied using linear regression analysis.

## Results

### Chronic MPLA delivery increases the phagocytic activity of microglial cells in the absence of a toxic inflammatory response in PLP-α-syn mice

**Fig. 1:**
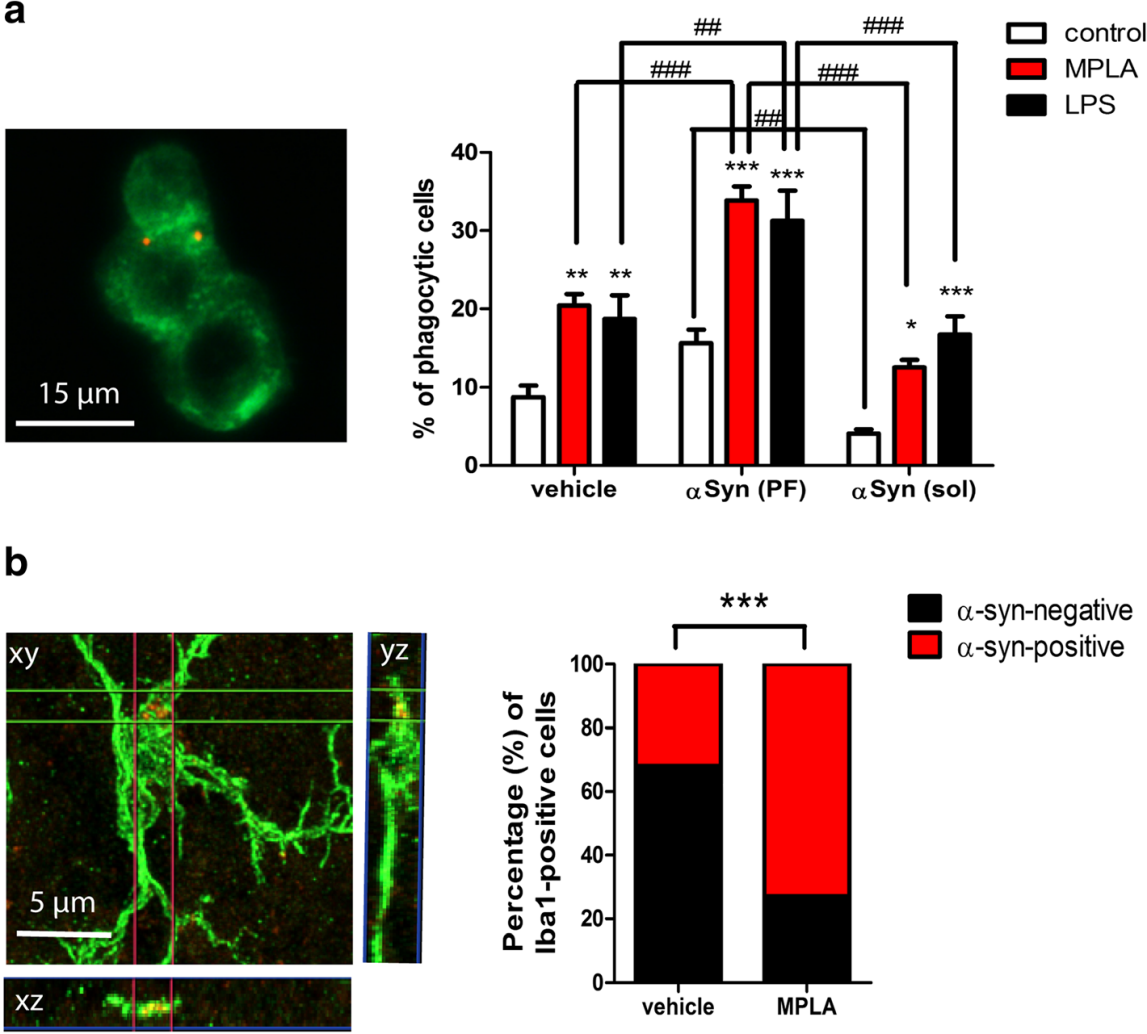
MPLA treatment increases the phagocytic activity of microglia. **a** Murine microglia were exposed to various treatments and phagocytic activity was measured by the incorporation of fluorescent beads. At baseline (vehicle treatment) both MPLA and LPS significantly increased the phagocytosis. Pre-exposure to fibrils of α-syn (αSyn (PF) treatment) further elevated the phagocytic activity of microglia treated with MPLA or LPS, whereas monomeric soluble α-syn (αSyn (sol) treatment) had no such effect. Data are means ± SEM, *n* = 4 per group. *p < 0.05, **p < 0.01, ***p < 0.001 compared to control. ^##^ *p* < 0.01, ^###^ *p* < 0.001. **b** Confocal image demonstrates the localization of human α-syn in the cytoplasm of Iba-1 positive microglial cell in a PLP-α-syn mouse treated with MPLA. Z-stack analysis showed more frequent detection of microglial cells incorporating α-syn after MPLA treatmentas compared to vehicle treated PLP-α-syn mice by Fischer’s exact test (***p < 0.001)

Extensive studies have characterized and documented the low toxicity immune response to MPLA linked to reduced release of pro-inflammatory cytokine and chemokine as compared to LPS [25]. We were interested in evaluating microglial phagocytic activity in response to MPLA as compared to LPS treatment in the presence or absence of monomeric or fibrillar α-syn. MPLA and LPS treatment both significantly augmented the phagocytic activity of microglial cells as compared to control cells without TLR4 agonist treatment. Moreover, microglial cells that received pre-exposure to fibrillar α-syn, but not soluble α-syn monomers showed a further significantly increased phagocytic activity (Fig. 1a). Following these in vitro results, we sought to identify microglial cells that were actively phagocytizing α-syn in vivo in the brains of PLP-α-syn mice receiving either vehicle or MPLA systemic treatment. Transgenic mice that received MPLA showed significantly higher percentage of α-syn phagocytizing microglia profiles (Fig. 1b).

**Fig. 2:**
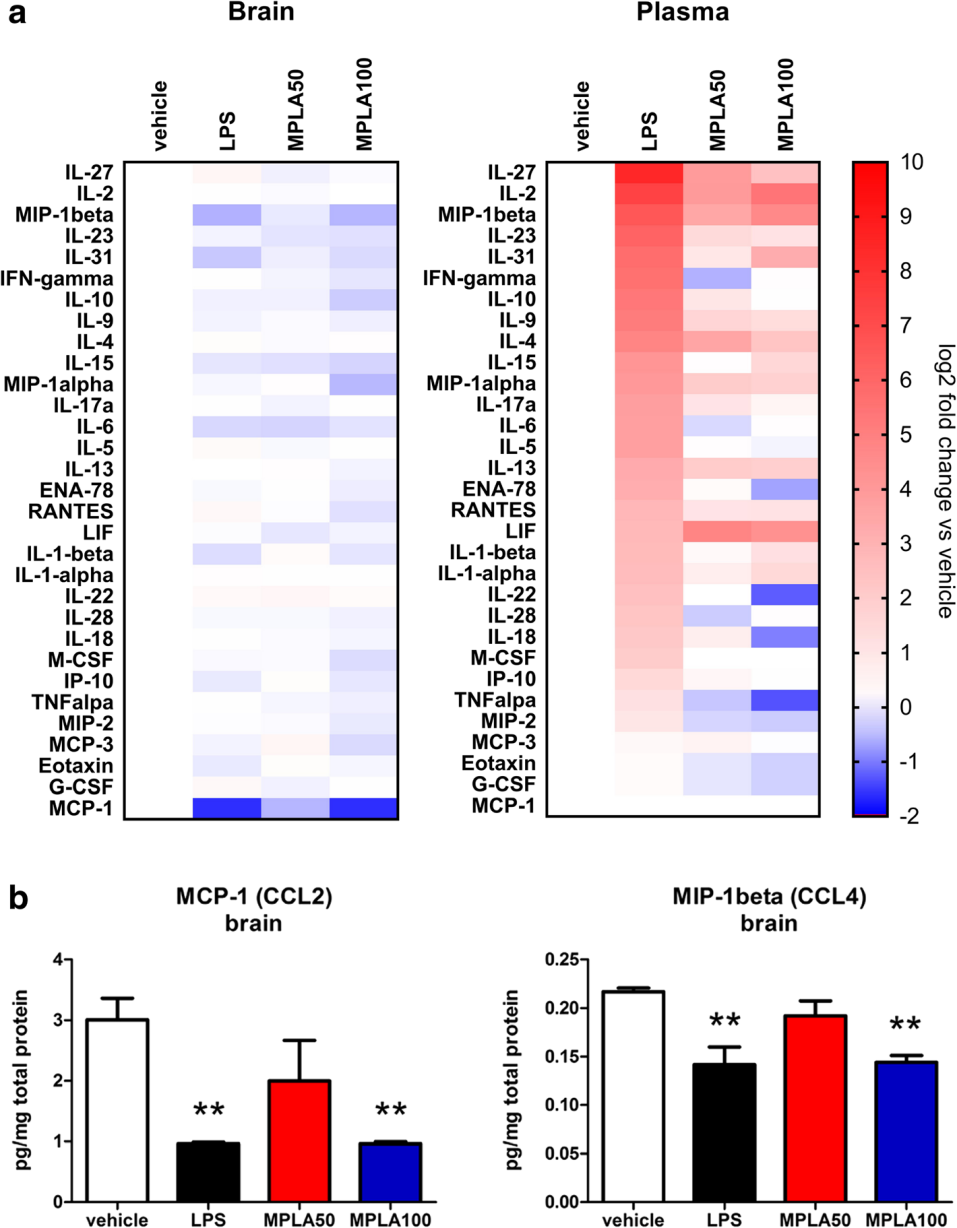
LPS, but not MPLA, causes systemic toxic inflammatory response in PLP-α-syn mice. **a** Heatmap demonstrates the overall log2 foldchange in the cytokine/chemokine levels in the brain and plasma as measured with the ProcartPlex Multiplex immunoassay 2 weeks after the last application of systemic low-dose LPS, MPLA50 or MPLA100. The following cytokine/chemokines of the 36-plex panel -GM-CSF, CXCL1, INF-α, IL-12p70, IL-3- were excluded from the analysis because no detectable levels were measured with this kit. **b** The detailed analysis of the cytokines/chemokines in the brains showed significant decrease of CCL2 and CCL4 after treatment with LPS or MPLA 100 μg. Data are means ± SEM, *n* = 4 per group. **p < 0.01 compared to vehicle treated mice

Next, we aimed to estimate whether chronic MPLA treatment can trigger a toxic inflammatory response as compared to chronic low dose LPS exposure in transgenic mice overexpressing human α-syn in oligodendrocytes. Noteworthy, the survival in both MPLA treatment groups as well as the vehicle treatment group was 100%, whereas the LPS treatment group had significantly increased acute mortality within 24 h after the LPS application with survival of 44% (comparison of survival curves with log rank test: chi-square = 35.27; *p* < 0.0001) suggesting acute toxic effect of LPS only. We measured cytokine/chemokine levels in the plasma and brain of PLP-α-syn mice receiving weekly i.p. injections of MPLA, LPS, or vehicle over a period of 12 weeks. Heatmap demonstrated different profile of the changes in the inflammatory status between long-term low dose LPS exposure and MPLA treatment groups (Fig. 2a). Specifically the chronic inflammatory response to LPS was characterized by increased levels of RANTES (CCL5, *p* < 0.05), MIP-1α (CCL3, *p* < 0.01), IL-10 (*p* < 0.05), IL-13 (*p* < 0.05), IL-27 (*p* < 0.05) and IP-10 (*p* < 0.05) detectable 2 weeks after the last LPS injection. In comparison, MPLA treatment in PLP-α-syn mice induced a very different inflammatory profile in the plasma. With the treatment of 50 μg MPLA in PLP-α-syn mice, increased levels of the anti-inflammatory cytokine LIF (*p* < 0.05) and of the chemokine MCP-3 (CCL7, *p* < 0.01) were detected. The higher dose of 100 μg MPLA resulted in a tendency towards increased levels of the anti-inflammatory cytokine LIF and significant decrease of the level of the pro-inflammatory Eotaxin (CCL11, *p* < 0.05). Altogether, the results suggest that in contrast to LPS chronic treatment, MPLA treatment has no systemic toxic inflammatory effects in the PLP-α-syn mice.

Analysis of the long-term neuroinflammatory response in the brains of PLP-α-syn mice revealed a downregulation of MIP-1β (CCL4), and MCP-1 (CCL2) following chronic treatment with TLR4 agonists (Fig. 2b).

### LPS but not MPLA treatment of PLP-α-syn mice causes redistribution of the α-syn species from soluble monomeric to pathological insoluble oligomeric α-syn

**Fig. 3:**
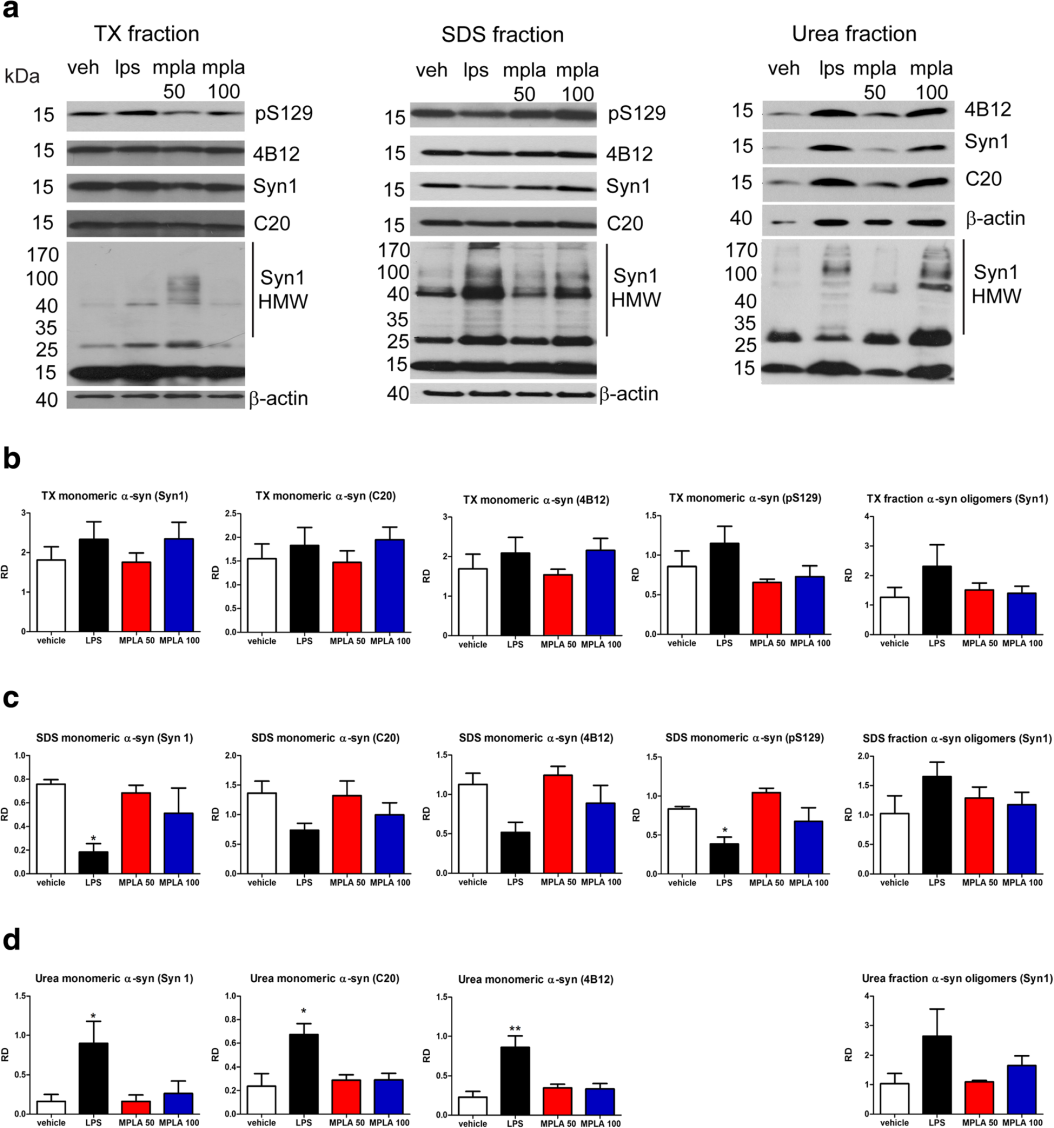
LPS, but not MPLA induces re-distribution of α-syn from soluble monomeric to insoluble and oligomeric species. **a** Brains of PLP-α-syn mice were lysed and three protein fractions were prepared – TX soluble fraction, SDS soluble fraction, and Urea fraction. Membranes were probed for four different α-syn antibodies (pS129, 4B12, Syn1, and C20). Relative density (RD) for monomeric α-syn (15 kDa) and oligomeric high molecular weight α-syn (HMW, over 30 kDa) was normalized to the intensity of the β-actin band (40 kDa). **b** No significant effects of the treatments on α-syn distribution were identified in the TX soluble fraction. **c** LPS treatment induced significant reduction of the SDS soluble monomeric α-syn and a tendency towards increase of the HMW SDS soluble α-syn species. MPLA treatment had no significant effect on the distribution of α-syn species in the SDS fraction. **d** In the urea fraction a significant increase of insoluble α-syn species was identified after LPS treatment, but not after MPLA systemic treatment of PLP-α-syn mice. Data are means ± SEM, *n* = 4 per group. * *p* < 0.05, ** *p* < 0.01 compared to vehicle treated mice

We were interested in how the changes in the neuroinflammatory milieu in the brains of PLP-α-syn mice after TLR4 agonist treatment may interfere with the α-syn pathology. No dynamic changes were identified in the TX fraction (Fig. 3a, b). In the SDS fraction there was a significant decrease of monomeric α-syn species (including pS129) and an apparent shift to more toxic oligomeric α-syn species after chronic LPS treatment, but not after MPLA treatment of PLP-α-syn mice (Fig. 3a, c). Finally, a significant increase of α-syn in the insoluble urea fraction was identified after LPS treatment, but not after MPLA treatment (Fig. 3a, d).

**Fig. 4:**
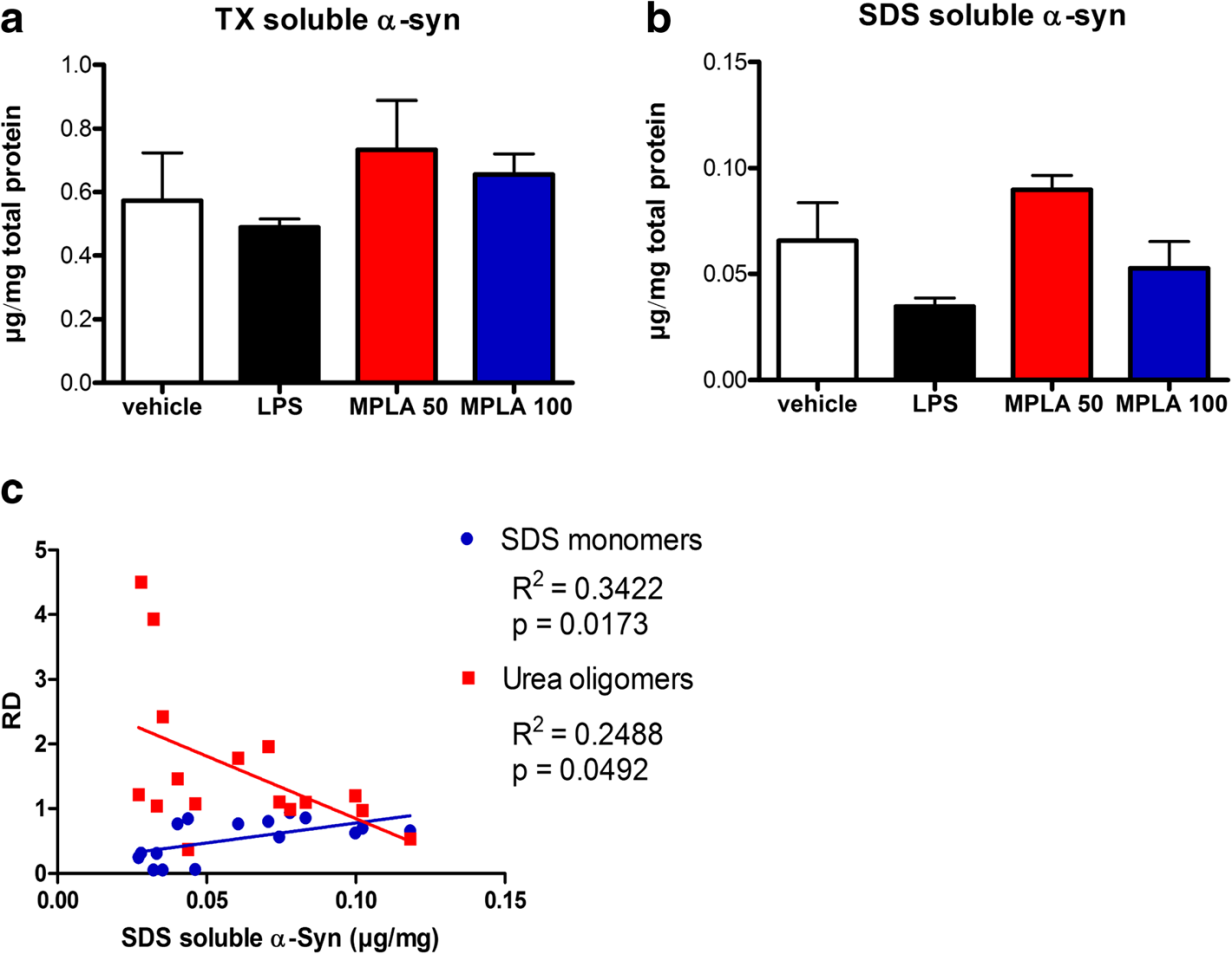
Absolute levels of soluble α-syn in the brains of PLP- α-syn mice remain unchanged after systemic chronic TLR4 agonist treatment. **a** TX soluble and **b** SDS soluble levels of α-syn were unaffected by the LPS or MPLA treatments. **c** The increase in the concentration of α-syn in the SDS fraction positively correlated with the increase in the RD of SDS-soluble monomeric species. In contrast, the higher concentration of α-syn measured in the SDS fraction by ELISA correlated with the lower RD of insoluble oligomeric α-syn species in the urea fraction, suggesting that the tendency of increased levels of α-syn after MPLA 50 in the SDS fraction may reflect the reduction of toxic insoluble oligomeric species after this treatment in PLP-α-syn mice. Data are means ± SEM, *n* = 4 per group

The absolute protein levels of α-syn after the 12 week long TLR4 agonist treatment were not significantly changed as assessed by ELISA in the soluble TX and SDS fractions (Fig. 4a, b). However we found a significant correlation between the absolute α-syn protein levels in the SDS fraction (not the TX fraction) as measured by ELISA and the reciprocal re-distribution from monomeric soluble to insoluble oligomeric species of α-syn measured by Western blot in the brains of PLP-α-syn mice after TLR4 agonist treatment (Fig. 4c).

### Chronic MPLA delivery ameliorates motor deficits and neuropathological lesions in PLP-α-syn mice

**Fig. 5:**
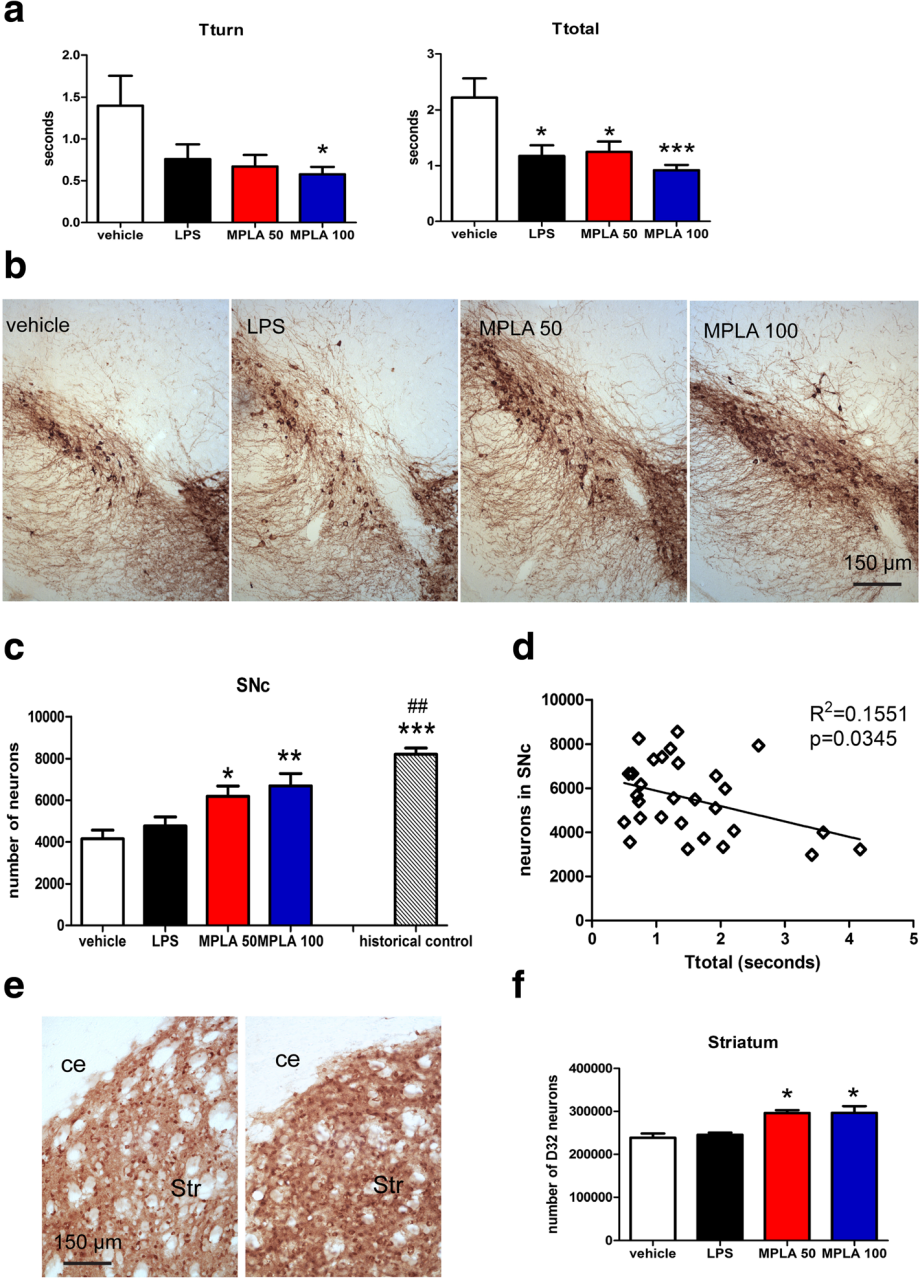
Systemic MPLA treatment of PLP-α-syn mice ameliorates the MSA-like phenotype. **a** Pole test showed significant improvement of the motor performance of PLP-α-syn after MPLA 100 μg treatment as compared to vehicle treated mice – both Tturn and Ttotal were significantly reduced. MPLA 50 μg and LPS treatment reduced T total but had no significant effect on Tturn. Data are means ± SEM, n_vehicle_ = 14, n_LPS_ = 7, n_MPLA50_ = 17, n_MPLA100_ = 16. **b** TH-immunohistochemistry was used to visualize dopaminergic neurons in SNc. **c** Stereology counts identified significant rescue of dopaminergic neurons in SNc after MPLA treatment (Data are means ± SEM, n_vehicle_ = 11, n_LPS_ = 3, n_MPLA50_ = 9, n_MPLA100_ = 7.* *p* < 0.05, ** *p* < 0.01 compared to vehicle treated mice). A historical healthy control group of age-, sex- and background-matched mice (*n* = 6) was added here to demonstrate the normal level of TH-positive neurons in SNc in non-tg mice (***p < 0.001 compared to vehicle treated PLP-α-syn mice, ##*p* < 0.01 compared to LPS treated PLP-α-syn mice). **d** The lower number of dopaminergic neurons in the transgenic SNc significantly correlated with the higher disability (increased Ttotal) in the pole test. **e** DARPP-32 immunoreactivity was used to define the number of GABAergic medium spiny neurons in the striatum (ce, capsula externa; Str, striatum. **f** Stereological analysis significant rescue of DARPP-32 neurons in the striatum after MPLA treatment (Data are means ± SEM, n_vehicle_ = 4, n_LPS_ = 3, n_MPLA50_ = 4, n_MPLA100_ = 4.* *p* < 0.05, compared to vehicle treated mice)

PLP-α-syn transgenic mice received weekly i.p. injections of MPLA (50 μg or 100 μg), vehicle, or LPS. Twelve weeks after the first injection the motor performance of the mice was tested in the pole test. The mice receiving MPLA (either dose) demonstrated significantly shorter times to orient downward (Tturn) and to descend the pole (Ttotal) compared to those receiving vehicle (Fig. 5a). In comparison, LPS treatment of PLP-α-syn mice resulted in significant motor improvement as measured by shortening in the total time necessary to climb down the pole but no significant change was seen in Tturn as compared to vehicle-treated animals.

The PLP-α-syn transgenic mouse is characterized by mild neuronal loss in the SNc [32] and striatum [33]. We were interested to find out if the observed motor improvement after TLR4 agonist treatment may be linked to neuronal rescue in SNc and striatum. We identified a significant preservation of dopaminergic neurons after MPLA treatment, but only a statistical tendency towards a higher number of TH-positive cells in SNc after LPS treatment as compared to vehicle-treated mice (Fig. 5b, c). The number of dopaminergic neurons in SNc mildly but significantly correlated with the motor performance of the PLP-α-syn mice in the pole test (Fig. 5d). Next, the analysis of DARPP-32-positive neurons in striatum indicated their rescue after MPLA treatment, but not after LPS treatment of PLP-α-syn mice (Fig. 5e, f).

**Fig. 6:**
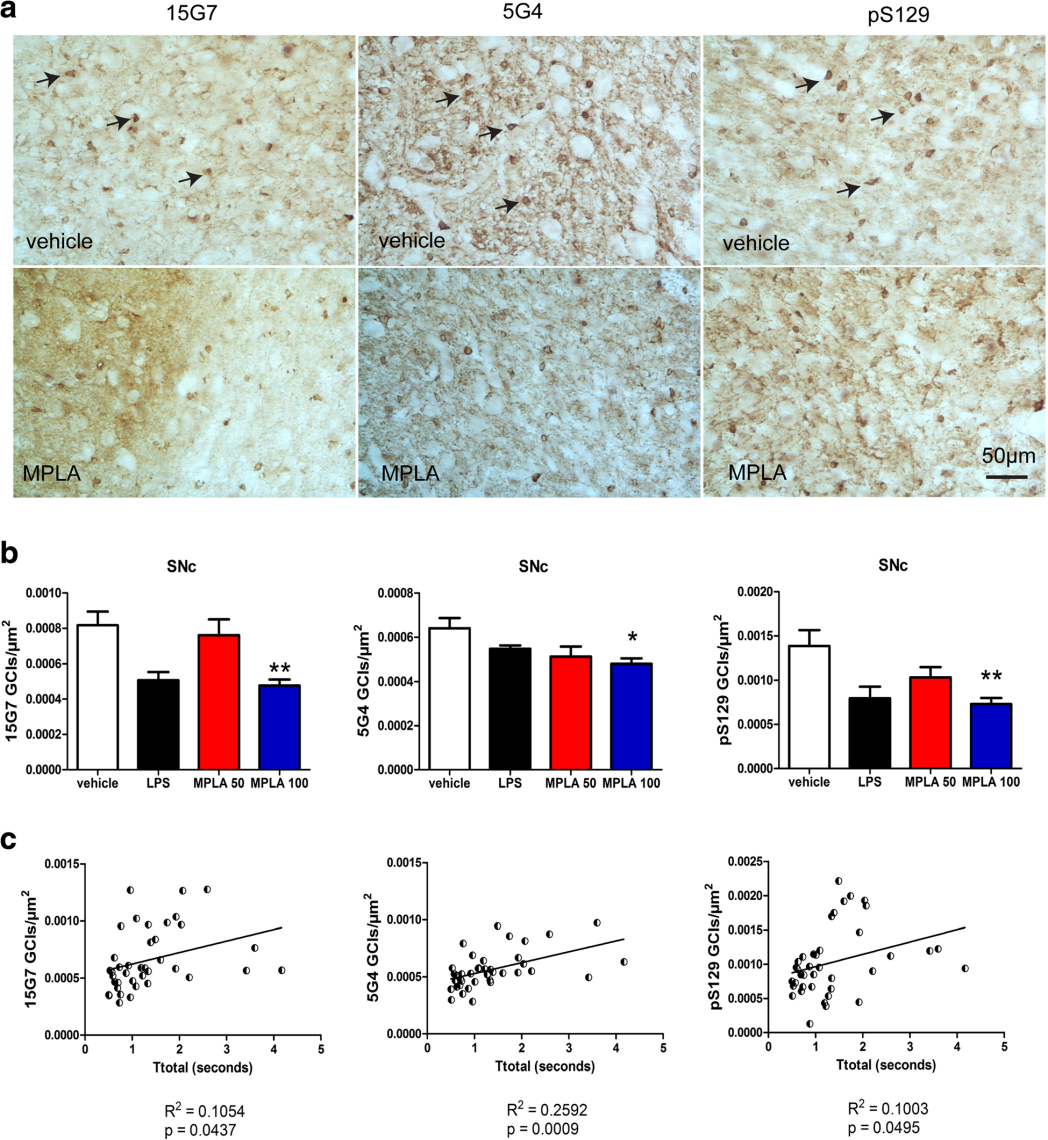
Systemic MPLA treatment of PLP-α-syn mice reduces the density of GCIs. **a** Three different antibodies to α-syn (15G7, 5G4, and pS129) were used to identify GCI-like accumulation of α-syn (arrows) in the brains of PLP-α-syn. **b** Image analysis demonstrated significant reduction of the density of GCIs in the SNc of PLP-α-syn mice treated with MPLA 100 μg as compared to vehicle-treated animals (Data are means ± SEM, n_vehicle_ = 13, n_LPS_ = 3, n_MPLA50_ = 13, n_MPLA100_ = 12.* *p* < 0.05, ** *p* < 0.01). **c** The decrease in the nigral GCI density significantly correlated with the motor improvement of the PLP-α-syn mice as measured by Ttotal of the pole test

To provide a region specific analysis of the α-syn intracellular accumulation in the SNc of PLP-α-syn mice after the different treatments, we analyzed the density of intracellular α-syn aggregates by immunohistochemistry. We identified a significant decrease of the density of α-syn intracellular accumulation (GCI-like pathology) in the SNc of PLP-α-syn mice treated with 100 μg MPLA as compared to vehicle treated animals by applying three different antibodies (Fig. 6a, b). Furthermore, the decrease of the GCIs density mildly but significantly correlated with the motor behavioral improvement in the pole test (Fig. 6c). Next we extended the analysis of GCI densities in other brain regions. Neither in the striatum, nor in the cerebellum we found significant effects of treatment on the GCI densities suggesting regional selectivity of the treatment effects (Additional file 1).

## Discussion

The data reported in the present study demonstrate that the selective TLR4 ligand MPLA is able to increase the phagocytic activity of microglia but does not trigger toxic inflammatory responses as generated by LPS after chronic 12 week treatment of PLP-α-syn transgenic mice. Furthermore, chronic systemic administration of MPLA does not trigger a shift from monomeric soluble towards oligomeric insoluble α-syn species in contrast to LPS treatment in PLP-α-syn mice. As a consequence of these effects, chronic MPLA therapy of PLP-α-syn mice significantly improved the motor deficits, which correlated with the rescue of dopaminergic neurons in the SNc and a decrease in the density of GCI-like inclusions in a region-specific manner in SNc.

The neuroinflammatory responses in neurodegenerative diseases are initiated as a natural intrinsic rescue response but after assuming a chronic course, they become potentially harmful [34–36]. Increasing evidence suggest that fine tuning of these responses may provide beneficial effects which therefore could serve as a therapeutic target. Rutherford and colleagues [37] suggested that the presence of LPS in preparations of α-syn that were exogenously injected in the mouse brain reduced the spread of α-syn pathology. Recently, Koller and colleagues also showed that inflammatory priming can have a beneficial role affecting the induction of α-syn pathology [38]. We previously demonstrated that genetic knock-out of the functional TLR4 in the PLP-α-syn mice, characterized by extraneuronal accumulation of α-syn in oligodendrocytes, results in deterioration of the motor deficits, aggravation of nigral neuronal loss, and elevation of the levels of α-syn in the brain [21]. The current experiments show that the chronic treatment with the low toxicity TLR4 ligand MPLA that selectively activates TLR4 but not TLR2, leads to improvement of the motor deficits of the PLP-α-syn mice associated with nigral and striatal neuroprotection. Whole-brain levels of soluble α-syn (TX and SDS fractions) were not changed after 12 week long treatment with MPLA of PLP-α-syn mice as measured by ELISA. Due to methodological limitations, the detection of levels of insoluble α-syn species in the urea fraction was not possible by ELISA, while Western immunoblot did not show any whole brain (but not addressing region specific) changes in soluble or insoluble species with MPLA. The lack of effects of MPLA treatment on the protein levels of α-syn measured by ELISA in contrast to the strong changes detected in the double transgenic PLP-α-syn/TLR4^−/−^ mouse [21] may be explained by either the different duration of the TLR4 activation/dysfunction (12 weeks treatment in the current study versus life-long deficiency of TLR4 after the genetic modification), or by region-specific changes that were masked when analyzing whole brain extracts. In support of the latter hypothesis, there was a significant region-specific reduction of the oligodendroglial aggregation of the α-syn in mice treated with the higher dose MPLA as identified by three different antibodies in immunohistochemical stainings in SNc of PLP-α-syn mice, but not in striatum or cerebellum. These region-specific effects of the MPLA treatment may be linked to different properties of the microglial cells and their propensity to execute TLR4-mediated signals in different brain regions. Indeed, SN-specific microglial phenotypes, neuroinflammatory events and vulnerability have been previously proposed [39–41] and may contribute to the reported here selectivity of effects. Furthermore, the decrease in the density of oligodendroglial α-syn inclusions in SNc mildly correlated with the motor performance of the animals further supporting the role of MPLA in modulating the oligodendroglial aggregation of α-syn in the PLP-α-syn model of MSA to ameliorate the motor dysfunction.

Another putative contributor to the beneficial effects of MPLA (especially its high dose) in the PLP-α-syn mice is the specific neuroinflammatory response observed after chronic treatment. We identified significant reduction of MCP-1 (CCL2) and MIP-1beta (CCL4) after 12 weeks of chronic treatment with 100 μg MPLA in the brains of PLP-α-syn mice. The monocyte chemoattractant protein-1 CCL2 and the macrophage inflammatory protein-1beta CCL4 are both produced by macrophages and characterized by their chemoattractant activity for monocytes, therefore representing important pro-inflammatory signaling components of the neuroinflammatory cascade [42]. A large body of experimental evidence supports the neuroprotective role of MCP-1 and MIP1-beta downregulation in various conditions [43–47]. Although a similar extent of reduction of CCL2 and CCL4 brain levels was observed in PLP-α-syn mice treated with LPS, the beneficial effects of LPS were limited due to several reasons. First, LPS treatment caused significant toxic inflammatory response in contrast to MPLA in the PLP-α-syn mouse model, similar to findings reported in other studies [48, 49]. Second, in contrast to MPLA, LPS had a strong effect on the re-distribution from non-toxic soluble monomeric to toxic insoluble oligomeric α-syn species in the brains of PLP-α-syn mice. This finding is supported by recent data reporting that LPS may directly (without the mediatory effect of neuroinflammatory responses) trigger the generation of distinct α-syn fibrils in vitro [50]. In summary, although LPS, like MPLA, is found to induce equally strong phagocytic activity in microglial cells and similar neuroinflammatory response is elicited after chronic systemic treatment, its beneficial effects are limited due to the deleterious effects on α-syn aggregation and the general pro-inflammatory response elicited in the plasma.

Finally, the results of the current study are well supported by the effects of MPLA systemic treatment in a mouse model of Alzheimer’s disease (AD). The beneficial effects of MPLA, but not LPS, on the cognitive function of AD mice was strongly linked with the enhanced phagocytic response of microglia and the reduction of the amyloid-β load in the brain of these mice [23]. Therefore, modulation of the innate immune response by MPLA may represent a possible therapeutic option for neurodegenerative diseases with abnormal protein deposition and features of prion-like cell-to-cell transmission, including α-synucleinopathies [13, 51, 52].

## Conclusions

Our data demonstrate the efficacy of a chronic TLR4 stimulation approach in a transgenic mouse model of MSA, a predominantly extraneuronal α-synucleinopathy, characterized by the atypical ectopic aggregation of the protein in oligodendrocytes. We present an in vivo proof of concept of the ability of the TLR4 agonist MPLA to ameliorate motor deficits and rescue nigral neurons in a transgenic mouse model of MSA through improved α-syn clearance in the absence of strong toxic inflammatory response. Since MPLA is clinically approved vaccine adjuvant [25], this makes it an even more intriguing and attractive candidate which, after in-depth analysis of treatment regimens in humans, may hold promise for the therapy of MSA and other α-synucleinopathies.

## Additional file

Additional file 1: Supplementary Table - Density of GCIs in the striatum and cerebellum. (DOCX 14 kb)

## Abbreviations

AD: Alzheimer’s disease
CCL: Chemokine (CC motif) ligand
DLB: Dementia with Lewy bodies
GCIs: Glial cytoplasmic inclusions
i.p.: Intraperitoneal
IL: Interleukin
IP-10: Interferon gamma-induced protein 10
LPS: Lipopolysaccharide
MCP-1: Monocyte chemoattractant protein 1
MIP-1: Macrophage inflammatory protein 1
MPLA: Monophosphoryl lipid A
MSA: Multiple system atrophy
PD: Parkinson’s disease
PLP: Proteolipid protein
RANTES: Regulated upon Activation, Normally T-expressed, and presumably Secreted
SDS: Sodium dodecyl sulfate
SNc: Substantia nigra pars compacta
TLR: Toll-like receptor
TX: Triton-X100
α-syn: α-synuclein

## References

[1] Spillantini MG, Schmidt ML, Lee VM, Trojanowski JQ, Jakes R, Goedert M (1997). Alpha-synuclein in Lewy bodies. Nature.

[2] Tu PH, Galvin JE, Baba M, Giasson B, Tomita T, Leight S (1998). Glial cytoplasmic inclusions in white matter oligodendrocytes of multiple system atrophy brains contain insoluble alpha-synuclein. Ann Neurol.

[3] Trojanowski JQ, Lee VM (2003). Parkinson's disease and related alpha-synucleinopathies are brain amyloidoses. Ann N Y Acad Sci.

[4] Spillantini MG, Crowther RA, Jakes R, Cairns NJ, Lantos PL, Goedert M (1998). Filamentous alpha-synuclein inclusions link multiple system atrophy with Parkinson's disease and dementia with Lewy bodies. Neurosci Lett.

[5] Fanciulli A, Wenning GK (2015). Multiple-system atrophy. N Engl J Med.

[6] Weinreb PH, Zhen W, Poon AW, Conway KA, Lansbury PT (1996). NACP, a protein implicated in Alzheimer's disease and learning, is natively unfolded. Biochemistry.

[7] Bertoncini CW, Jung YS, Fernandez CO, Hoyer W, Griesinger C, Jovin TM (2005). Release of long-range tertiary interactions potentiates aggregation of natively unstructured alpha-synuclein. Proc Natl Acad Sci U S A.

[8] Bertoncini CW, Fernandez CO, Griesinger C, Jovin TM, Zweckstetter M (2005). Familial mutants of alpha-synuclein with increased neurotoxicity have a destabilized conformation. J Biol Chem.

[9] Braak H, Del TK, Rub U, De Vos RA, Jansen Steur EN, Braak E (2003). Staging of brain pathology related to sporadic Parkinson's disease. Neurobiol Aging.

[10] Luk KC, Kehm V, Carroll J, Zhang B, O'Brien P, Trojanowski JQ (2012). Pathological alpha-synuclein transmission initiates Parkinson-like neurodegeneration in nontransgenic mice. Science.

[11] Watts JC, Giles K, Oehler A, Middleton L, Dexter DT, Gentleman SM (2013). Transmission of multiple system atrophy prions to transgenic mice. Proc Natl Acad Sci U S A.

[12] Lee HJ, Suk JE, Patrick C, Bae EJ, Cho JH, Rho S (2010). Direct transfer of alpha-synuclein from neuron to astroglia causes inflammatory responses in synucleinopathies. J Biol Chem.

[13] Lee HJ, Bae EJ, Lee SJ (2014). Extracellular alpha--synuclein-a novel and crucial factor in Lewy body diseases. Nat Rev Neurol.

[14] Fellner L, Jellinger KA, Wenning GK, Stefanova N (2011). Glial dysfunction in the pathogenesis of alpha-synucleinopathies: emerging concepts. Acta Neuropathol.

[15] Gordon S (2002). Pattern recognition receptors: doubling up for the innate immune response. Cell.

[16] Letiembre M, Liu Y, Walter S, Hao W, Pfander T, Wrede A (2009). Screening of innate immune receptors in neurodegenerative diseases: a similar pattern. Neurobiol Aging.

[17] Stefanova N, Reindl M, Neumann M, Kahle PJ, Poewe W, Wenning GK (2007). Microglial activation mediates neurodegeneration related to oligodendroglial alpha-synucleinopathy: implications for multiple system atrophy. Mov Disord.

[18] Brudek T, Winge K, Agander TK, Pakkenberg B (2013). Screening of toll-like receptors expression in multiple system atrophy brains. Neurochem Res.

[19] Beraud D, Maguire-Zeiss KA (2012). Misfolded alpha-synuclein and toll-like receptors: therapeutic targets for Parkinson's disease. Parkinsonism Relat Disord.

[20] Fellner L, Irschick R, Schanda K, Reindl M, Klimaschewski L, Poewe W (2013). Toll-like receptor 4 is required for alpha-synuclein dependent activation of microglia and astroglia. Glia.

[21] Stefanova N, Fellner L, Reindl M, Masliah E, Poewe W, Wenning GK (2011). Toll-like receptor 4 promotes alpha-synuclein clearance and survival of nigral dopaminergic neurons. Am J Pathol.

[22] Johnson DA, Keegan DS, Sowell CG, Livesay MT, Johnson CL, Taubner LM (1999). 3-O-Desacyl monophosphoryl lipid a derivatives: synthesis and immunostimulant activities. J Med Chem.

[23] Michaud JP, Halle M, Lampron A, Theriault P, Prefontaine P, Filali M (2013). Toll-like receptor 4 stimulation with the detoxified ligand monophosphoryl lipid a improves Alzheimer's disease-related pathology. Proc Natl Acad Sci U S A.

[24] Sangar VC, Ghongane B, Mathur G (2016). Development of human Papillomavirus (HPV) vaccines: a review of literature and clinical update. Rev Recent Clin Trials.

[25] Casella CR, Mitchell TC (2008). Putting endotoxin to work for us: monophosphoryl lipid a as a safe and effective vaccine adjuvant. Cell Mol Life Sci.

[26] Cluff CW (2010). Monophosphoryl lipid a (MPL) as an adjuvant for anti-cancer vaccines: clinical results. Adv Exp Med Biol.

[27] Rego A, Viana SD, Ribeiro CA, Rodrigues-Santos P, Pereira FC (2016). Monophosphoryl lipid-a: a promising tool for Alzheimer's disease toll. J Alzheimers Dis.

[28] Kahle PJ, Neumann M, Ozmen L, Muller V, Jacobsen H, Spooren W (2002). Hyperphosphorylation and insolubility of alpha-synuclein in transgenic mouse oligodendrocytes. EMBO Rep.

[29] Stefanova N, Kaufmann WA, Humpel C, Poewe W, Wenning GK (2012). Systemic proteasome inhibition triggers neurodegeneration in a transgenic mouse model expressing human alpha-synuclein under oligodendrocyte promoter: implications for multiple system atrophy. Acta Neuropathol.

[30] Volpicelli-Daley LA, Luk KC, Lee VM (2014). Addition of exogenous alpha-synuclein preformed fibrils to primary neuronal cultures to seed recruitment of endogenous alpha-synuclein to Lewy body and Lewy neurite-like aggregates. Nat Protoc.

[31] Emmanouilidou E, Elenis D, Papasilekas T, Stranjalis G, Gerozissis K, Ioannou PC (2011). Assessment of alpha-synuclein secretion in mouse and human brain parenchyma. PLoS One.

[32] Stefanova N, Reindl M, Neumann M, Haass C, Poewe W, Kahle PJ (2005). Oxidative stress in transgenic mice with oligodendroglial alpha-synuclein overexpression replicates the characteristic neuropathology of multiple system atrophy. Am J Pathol.

[33] Stefanova N, Kuen J, Borm C, Poewe W, Wenning GK (2014). Age-related analysis of striatonigral degeneration and olivopontocerebellar atrophy in the PLP-a-synuclein transgenic mouse model of MSA. Mov Disord.

[34] Fellner L, Stefanova N (2013). The role of glia in alpha-synucleinopathies. Mol Neurobiol.

[35] Bruck D, Wenning GK, Stefanova N, Fellner L (2016). Glia and alpha-synuclein in neurodegeneration: a complex interaction. Neurobiol Dis.

[36] Tejera D, Heneka MT (2016). Microglia in Alzheimer's disease: the good, the bad and the ugly. Curr Alzheimer Res.

[37] Rutherford NJ, Sacino AN, Brooks M, Ceballos-Diaz C, Ladd TB, Howard JK (2015). Studies of lipopolysaccharide effects on the induction of alpha-synuclein pathology by exogenous fibrils in transgenic mice. Mol Neurodegener.

[38] Koller EJ, Brooks MM, Golde TE, Giasson BI, Chakrabarty P (2017). Inflammatory pre-conditioning restricts the seeded induction of alpha-synuclein pathology in wild type mice. Mol Neurodegener.

[39] Tansey MG, Goldberg MS (2010). Neuroinflammation in Parkinson's disease: its role in neuronal death and implications for therapeutic intervention. Neurobiol Dis.

[40] Halliday GM, Stevens CH (2011). Glia: initiators and progressors of pathology in Parkinson's disease. Mov Disord.

[41] Doorn KJ, Breve JJ, Drukarch B, Boddeke HW, Huitinga I, Lucassen PJ (2015). Brain region-specific gene expression profiles in freshly isolated rat microglia. Front Cell Neurosci.

[42] Cartier L, Hartley O, Dubois-Dauphin M, Krause KH (2005). Chemokine receptors in the central nervous system: role in brain inflammation and neurodegenerative diseases. Brain Res Brain Res Rev.

[43] Yang G, Meng Y, Li W, Yong Y, Fan Z, Ding H (2011). Neuronal MCP-1 mediates microglia recruitment and neurodegeneration induced by the mild impairment of oxidative metabolism. Brain Pathol.

[44] Yuan B, Shi H, Zheng K, Su Z, Su H, Zhong M (2017). MCP-1-mediated activation of microglia promotes white matter lesions and cognitive deficits by chronic cerebral hypoperfusion in mice. Mol Cell Neurosci.

[45] Yang Z, Wang J, Yu Y, Li Z (2016). Gene silencing of MCP-1 prevents microglial activation and inflammatory injury after intracerebral hemorrhage. Int Immunopharmacol.

[46] Liu C, Cui G, Zhu M, Kang X, Guo H (2014). Neuroinflammation in Alzheimer's disease: chemokines produced by astrocytes and chemokine receptors. Int J Clin Exp Pathol.

[47] Wu YP, Proia RL (2004). Deletion of macrophage-inflammatory protein 1 alpha retards neurodegeneration in Sandhoff disease mice. Proc Natl Acad Sci U S A.

[48] Bohannon JK, Hernandez A, Enkhbaatar P, Adams WL, Sherwood ER (2013). The immunobiology of toll-like receptor 4 agonists: from endotoxin tolerance to immunoadjuvants. Shock.

[49] Ruchaud-Sparagano MH, Mills R, Scott J, Simpson AJ (2014). MPLA inhibits release of cytotoxic mediators from human neutrophils while preserving efficient bacterial killing. Immunol Cell Biol.

[50] Kim C, Lv G, Lee JS, Jung BC, Masuda-Suzukake M, Hong CS (2016). Exposure to bacterial endotoxin generates a distinct strain of alpha-synuclein fibril. Sci Rep.

[51] Hasegawa M, Nonaka T, Masuda-Suzukake M. Prion-like mechanisms and potential therapeutic targets in neurodegenerative disorders. Pharmacol Ther. 2016; 10.1016/j.pharmthera.2016.11.010 27916654

[52] Goedert M, Masuda-Suzukake M, Falcon B (2017). Like prions: the propagation of aggregated tau and alpha-synuclein in neurodegeneration. Brain.

