# Supplementary material for: Toll-like receptor 4 stimulation with monophosphoryl lipid A ameliorates motor deficits and nigral neurodegeneration triggered by extraneuronal α-synucleinopathy
Source: Mol Neurodegener. 2017 Jul 4;12:52. doi: 10.1186/s13024-017-0195-7 (PMC5496237; doi:10.1186/s13024-017-0195-7)
Supplement: Additional file 1: — Supplementary Table - Density of GCIs in the striatum and cerebellum. (DOCX 14 kb) [file 13024_2017_195_MOESM1_ESM.docx]

| GCIs/µm^2^  (e-005) | Striatum | | | | |
| --- | --- | --- | --- | --- | --- |
|  | Vehicle (n=6) | LPS (n=3) | MPLA50 (n=6) | MPLA100 (n=8) | P |
| 15G7 | 29.1±10.6 | 31.8±7.7 | 30.1±11.7 | 25.4±5.1 | 0.6681 |
| pS129 | 33.4±4.6 | 49.4±8.1 | 37.0±16.1 | 36.5±8.0 | 0.1992 |
| 5G4 | 35.7±11.6 | 33.0±5.9 | 31.3±3.1 | 30.0±4.1 | 0.4934. |
| GCIs/µm^2^  (e-005) | Cerebellum | | | | |
| 15G7 | 48.7±11.8 | 53.9±15.1 | 52.7±13.1 | 42.6±8.0 | 0.3319 |
| pS129 | 61.3±20.3 | 83.2±2.7 | 64.6±25.7 | 64.4±24.2 | 0.5622 |
| 5G4 | 50.1±15.7 | 47.1±10.9 | 48.7±10.9 | 42.1±8.7 | 0.5989 |

Additional file 1: Table

The density of GCIs in the striatum and cerebellum as labelled by three anti-α-syn antibodies (15G7, pS129, and 5G4) in the various treatment groups did not differ significantly.
